# A Strong Deletion Bias in Nonallelic Gene Conversion

**DOI:** 10.1371/journal.pgen.1002508

**Published:** 2012-02-16

**Authors:** Raquel Assis, Alexey S. Kondrashov

**Affiliations:** 1 Department of Integrative Biology, Center for Theoretical Evolutionary Genomics, University of California Berkeley, Berkeley, California, United States of America; 2 Department of Ecology and Evolutionary Biology, Center for Computational Medicine and Bioinformatics, University of Michigan, Ann Arbor, Michigan, United States of America; 3 Life Sciences Institute, University of Michigan, Ann Arbor, Michigan, United States of America; Aarhus University, Denmark

## Abstract

Gene conversion is the unidirectional transfer of genetic information between orthologous (allelic) or paralogous (nonallelic) genomic segments. Though a number of studies have examined nucleotide replacements, little is known about length difference mutations produced by gene conversion. Here, we investigate insertions and deletions produced by nonallelic gene conversion in 338 *Drosophila* and 10,149 primate paralogs. Using a direct phylogenetic approach, we identify 179 insertions and 614 deletions in *Drosophila* paralogs, and 132 insertions and 455 deletions in primate paralogs. Thus, nonallelic gene conversion is strongly deletion-biased in both lineages, with almost 3.5 times as many conversion-induced deletions as insertions. In primates, the deletion bias is considerably stronger for long indels and, in both lineages, the per-site rate of gene conversion is orders of magnitudes higher than that of ordinary mutation. Due to this high rate, deletion-biased nonallelic gene conversion plays a key role in genome size evolution, leading to the cooperative shrinkage and eventual disappearance of selectively neutral paralogs.

## Author Summary

Gene conversion is a process whereby a DNA sequence is copied from one segment of the genome (donor) to another (recipient), resulting in the replacement, insertion, or deletion of a DNA sequence in the recipient. This exchange is facilitated by the high sequence similarity of the two segments, which is due to their evolutionary relationship. Here, we study insertions and deletions produced by gene conversion between paralogs, segments related by DNA duplication events. By comparing paralog sequences in multiple species of fruit flies and primates, we find that deletions occur more than three times as frequently as insertions. We also discover that the rate of gene conversion between paralogs is quite high. The deletion bias and high rate of this process causes paralogs to shrink cooperatively and eventually be eliminated from the genome. Because of the abundance of paralogs in animal genomes, this phenomenon can lead to a significant reduction in genome size. Therefore, our finding enhances our understanding of the forces that lead to changes in genome size during evolution.

## Introduction

All genomes contain similar DNA segments. In diploids, such segments can be classified as orthologs or paralogs. Orthologs, or allelic segments, are paired copies located at the same genomic loci on maternal and paternal chromosomes. In contrast, paralogs, or nonallelic segments, are found at different genomic loci and can have any copy number, in which each copy is derived from an ancestral sequence via gene duplication [1].

The sequences of related DNA segments can diverge via ordinary mutation or converge via gene conversion. Ordinary mutation is generally AT-biased for nucleotide replacements [2]–[4] and deletion-biased for length difference mutations [5]. A number of studies have examined nucleotide replacements produced by allelic and nonallelic gene conversion, some of which have uncovered a GC bias [6]–[9]. Here, we explore length difference mutations produced by nonallelic gene conversion.

**Figure 1:**
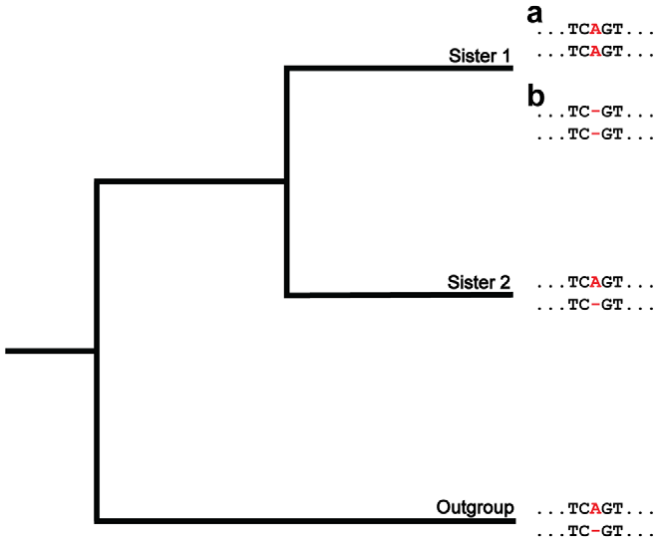
A phylogenetic approach for detecting insertions and deletions produced by nonallelic gene conversion. Depicted is a hypothetical multiple alignment for pairs of paralogs in two sisters and an outgroup. The two sequences for each species represent a pair of paralogs, and the position of interest is colored in red. At this position, a length difference (A/−) exists between the paralogs in sister 2 and the outgroup (ancestral state). In the lineage of sister 1, an insertion (a) or deletion (b) of a nucleotide occurs in one paralog. Because these events result in the paralogs having matching states (A/A or −/−), they are consistent with gene conversion.

In contrast to orthologs, paralogs have their own independent long-term phylogenies, making it possible to apply a direct phylogenetic approach to study their coevolution by gene conversion (Figure 1). For this approach, we utilized multiple alignments of pairs of paralogs in two sister species and an outgroup. First, we ascertained all cases in which, at a particular alignment position, there was an ancestral length difference between the paralogs, *i.e.*, the difference was present in one sister and in the outgroup. We then examined orthologous positions in the other sister and identified those cases for which there was no length difference between paralogs. Elimination of a length difference was due to an insertion if one paralog acquired an additional nucleotide(s) at that position, and was due to a deletion if it lost a nucleotide(s) at that position. If the event resulted in the paralogs having identical states at the affected position, it was consistent with gene conversion. A benefit of this approach is that it assumes nothing about the process or biases of ordinary mutation, because an ancestral length difference between paralogs can be caused by either an insertion or a deletion. Moreover, only a small proportion of indels identified using this approach were due to either ordinary mutation or sequencing errors (see Text S1).

## Results/Discussion

**Figure 2:**
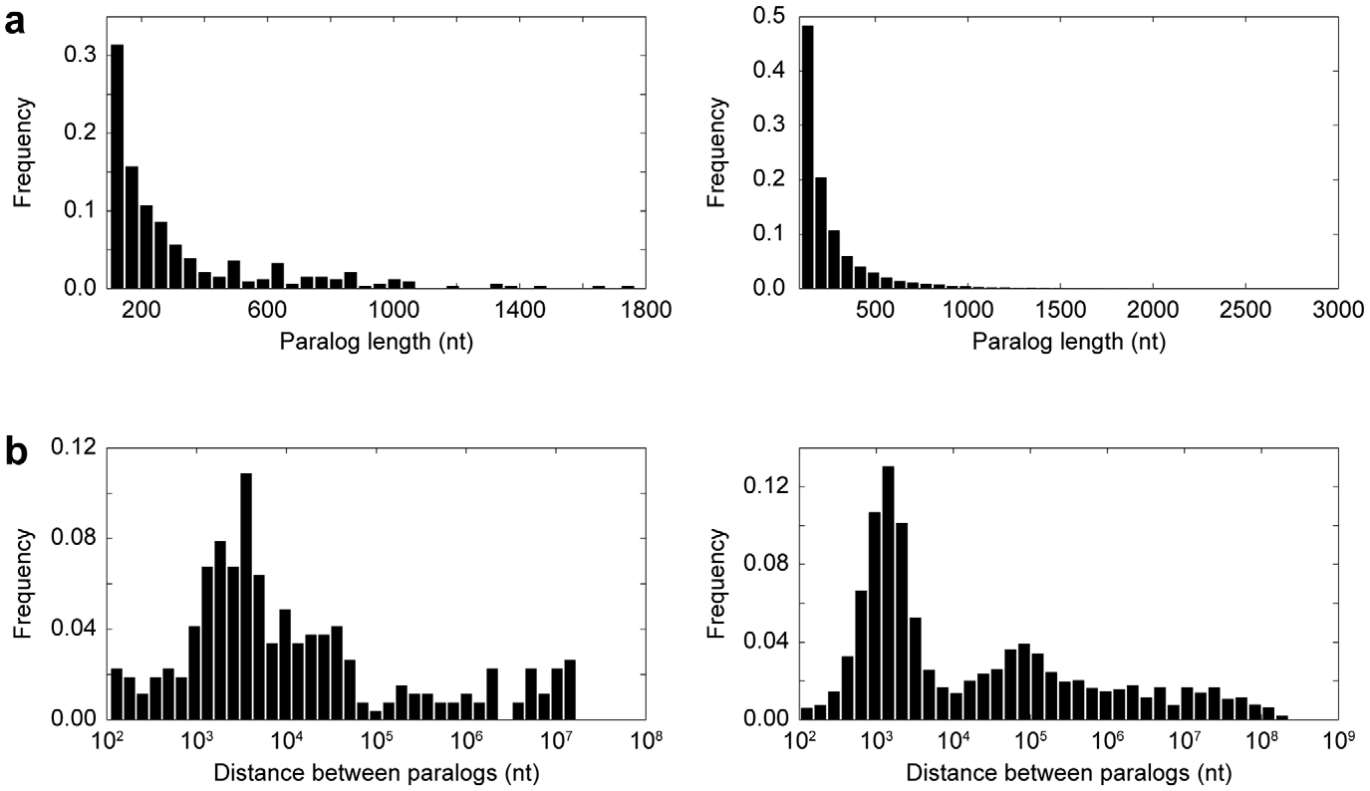
Properties of paralogs. (a) Distribution of paralog sequence lengths in *Drosophila* (left) and primates (right). (b) Distribution of distances between pairs of paralogs located on the same chromosome in *Drosophila* (left) and primates (right). Distances are plotted on a log scale.

Since our approach required that paralogs be present in the genomes of triplets of closely-related species, we chose to study gene conversion in *Drosophila* and primate lineages, for which whole-genome sequences of multiple close species are available. For *Drosophila*, we used *D. melanogaster* and *D. simulans* as sister species and *D. yakuba* as an outgroup, and for primates, we used human and chimpanzee as sisters and orangutan as an outgroup. We obtained 338 (199 coding) and 10,149 (1,740 coding) pairs of paralogs that are present in all three species of *Drosophila* and primates, respectively (Figure 2). Of these, 267 are intra-chromosomal in *Drosophila*, and 5,997 are intra-chromosomal in primates. Our phylogenetic analysis revealed that 101 *Drosophila* paralogs and 400 primate paralogs underwent gene conversion during the evolutionary timeframes considered. A general prediction of nonallelic gene conversion is that a pair of paralogs should be more similar in the genome of the species in which they underwent conversion than in the genomes of the other sister or outgroup. As expected, 95 paralogs in *Drosophila*, and 385 paralogs in primates display this trend.

**Figure 3:**
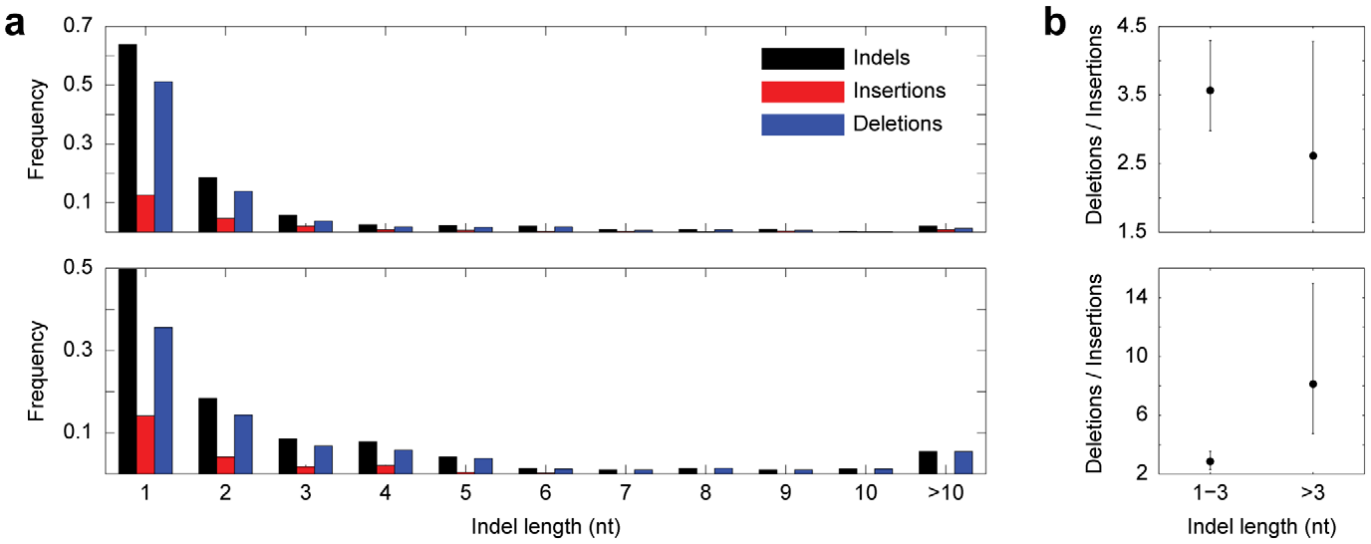
Indels consistent with gene conversion. (a) Length distributions of all indels, insertions, and deletions in *Drosophila* (top) and primates (bottom). (b) Strength of deletion bias as a function of indel length in *Drosophila* (top) and primates (bottom). Error bars represent confidence limits from binomial sign tests (see Methods).

Within our set of paralogs, we identified 179 insertions and 614 deletions consistent with gene conversion in *Drosophila*, and 132 insertions and 455 deletions consistent with gene conversion in primates (Figure 3a). Thus, there were ∼3.4 times as many deletions as insertions in both lineages, which was highly significant (*p*<0.0001). In primates, we found that the deletion bias was substantially larger for long than for short indels (Figure 3b). Exclusion of indels that occurred in coding regions, which were rare (45 in *Drosophila* and 27 in primates), did not alter the deletion bias in either lineage, implying that selection on coding paralogs did not affect the overall deletion/insertion ratios observed.

One concern of our parsimony-based approach is homoplasy, which would cause us to misidentify two ordinary mutation-induced indels of the same type, one in sister 2 and one in the outgroup, as one conversion-consistent indel in sister 1 (see Figure 1). However, homoplasy is unlikely in our dataset for several reasons. First, in contrast to nucleotide replacements, identical independent indel mutation events are rare [10], [11]. Though one group did uncover evidence of homoplastic indels [12], their analysis compared orthologs in very distantly-related species, the closest sisters being human and mouse, which have an average synonymous substitution rate, or *K*_s_, of ∼0.77 [13]. In contrast, the *K*_s_ between *D. melanogaster* (*D. simulans*) and *D. yakuba* is 0.23 (0.21) [14], and the *K*_s_ between human (chimpanzee) and orangutan is 0.03 (0.03) [15]. Thus, there was much less time for multiple independent indel mutations to occur. Second, paralogs in our dataset do not contain any satellite sequences, which are prone to homoplastic mutations [16]–[19], and are minimally repetitive in general (1.69% of *Drosophila* sequences, and 1.51% of primate sequences). Third, paralogs containing conversion-consistent indels follow the same genomic distribution as the entire set of paralogs (Figure S1), making it unlikely that spatial variation in mutation rate led to the observed patterns. Finally, most conversion events occurred between noncoding paralogs, which are less likely to be under selection for similar function, and also are not as limited as coding paralogs in the types of indels (nucleotide content, size) that can occur.

Even if homoplasy did occur, it would much more likely cause misidentifications of conversion-consistent insertions than deletions, leading to downward biases of deletion/insertion ratios. This is because, for one, homplastic events resembling conversion-consistent deletions require two insertions, which have lower mutation rates than insertions. Additionally, these insertions must be identical in sequence. In the case of single nucleotide insertions, there is a ¼ probability of the second insertion being identical to the first, and this probability rapidly decreases with increasing insertion sequence length.

We next estimated the rate of nonallelic gene conversion in *Drosophila* and primates. For primates, we performed a simple calculation. There are 28,701 sites at which there was an ancestral length difference between paralogs. Conversion-consistent indels occurred at 587 of these sites, resulting in ∼0.02 indels per site. For *Drosophila*, a more complex estimate was needed. There were 793 conversion-consistent indels that occurred at 960 possible sites, resulting in 0.83 indels per site. Due to this high proportion, it was necessary to correct for multiple conversion events per site. If we assume that gene conversion is a Poisson process, like ordinary mutation, the mean number of events per site is −*ln*(1−0.83), or ∼1.8. Because the number of events per site was much smaller in primates than in *Drosophila*, applying this correction to primate conversion events did not alter the original rate estimate.

Strong sequence similarity of paralogs is associated with high gene conversion rate [20]. To study this phenomenon, we computed Spearman correlation coefficients between paralog similarities and the number of gene conversion indels identified. In *Drosophila*, similarity was indeed positively correlated with gene conversion rate (ρ = 0.35; *p* = 2.3×10^−11^). However, in primates, there was instead a very weak negative relationship between similarity and gene conversion rate (ρ = −0.04; *p* = 2.45×10^−5^). Though it is possible that gene conversion rate does not increase with similarity in primates, it is more likely that this result is due to properties of our data. In particular, the primates compared in this study are an order of magnitude more closely related than the *Drosophila*: the *K*_s_ between human and chimpanzee is 0.01 [15], whereas the *K*_s_ between *D. melanogaster* and *D. simulans* is 0.11 [14]. Thus, similarities between primate paralogs tend to be higher and have a narrower range, making it difficult to assess the relationship between similarity and gene conversion rate in primates. Additionally, our data do not reflect “invisible” gene conversion events, or those that occurred between two identical sequences, which are likely to be more prevalent in primates due to the higher similarities of paralogs. The absence of such cases may have also affected our calculation in *Drosophila*, producing an underestimate of the correlation between sequence similarity and nonallelic gene conversion rate.

Physical distance between paralogs is also believed to influence gene conversion rate, with paralogs separated by smaller distances hypothesized to undergo faster gene conversion [21]. To study this relationship, we calculated end-to-end distances between pairs of paralogs located on the same chromosomes and computed Spearman coefficients between these distances and numbers of gene conversion indels detected after correcting for divergence between paralogs. In both lineages, there was a very weak positive correlation (ρ = 0.07 for *Drosophila*, ρ = 0.05 for primates), which was statistically significant only in primates (*p* = 2.79×10^−5^). These findings agree with those of McGrath *et al.* [22], who pointed out that negative correlations between distance and gene conversion rate are likely due to the fact that adjacent paralogs tend to be more similar to each other because of their recent ancestry [23].

Though a higher meiotic recombination rate is associated with elevated rates of allelic gene conversion, the relationship between recombination rate and nonallelic gene conversion is unclear. To investigate the potential relationship between these two parameters, we computed Spearman correlation coefficients between mean recombination rates for pairs of paralogs and numbers of gene conversion indels ascertained. Interestingly, in *Drosophila*, meiotic recombination rate was negatively correlated with nonallelic gene conversion rate (ρ = −0.22; *p* = 7.38×10^−5^). In contrast, we did not detect a correlation between these parameters in primates (ρ = 0.005; *p* = 0.63).

Next, we calculated the indel mutation rate in *Drosophila* and primates. To do this, we applied a parsimony-based approach to identify indels produced by ordinary mutation in each lineage. In *Drosophila*, we observed 202 indels produced by ordinary mutation. Of these indels, 18 were insertions and 184 were deletions, resulting in a deletion bias of ∼10∶1, which is consistent with previous estimates [24]. The target size for such mutations was half the length of all paralogs, which was 104,478 nt. However, as with our conversion analysis, we assumed indel mutations did not occur at the ends of sequences. Subtracting 338 positions, the sequence length along which indels could occur was 104,140 nt, resulting in ∼1.94×10^−3^ indel mutations per site in *Drosophila*. In primates, we observed 1,095 indels (533 insertions and 562 deletions) within a total sequence length of 2,448,263 nt, giving a rate of ∼4.47×10^−4^ indel mutations per site in primates.

Comparison of the rates of indels produced by nonallelic gene conversion and ordinary mutation revealed that nonallelic gene conversion is ∼927.8 times faster in *Drosophila* and ∼44.7 times faster in primates. This rapid deletion-biased process has a significant effect on genome size evolution. To illustrate this hypothesis, let us consider the life cycle of a length difference mutation within two paralogs. First, ordinary mutation introduces an insertion or deletion in one paralog. Then, deletion-biased gene conversion occurs between the paralogs. If the initial mutation was an insertion, it is removed. Otherwise, the deletion is transmitted to the second paralog, *i.e.*, fixed within the pair of paralogs. In the absence of selection, this process results in the cooperative shrinkage of these paralogous sequence segments.

**Figure 4:**
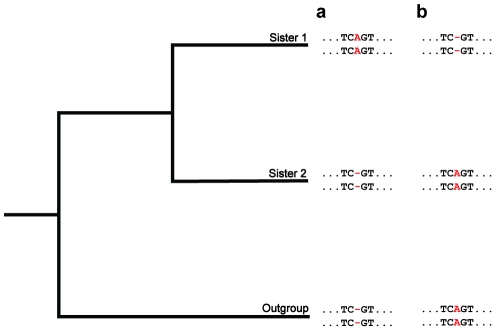
A phylogenetic approach for detecting fixed indels. Depicted are hypothetical multiple alignments for pairs of paralogs in two sisters and an outgroup. The two sequences for each species represent a pair of paralogs, and the position of interest is colored in red. (a and b) At this position, both paralogs have identical lengths in sister 2 and the outgroup (ancestral state). In the lineage of sister 1, identical insertions (a) or deletions (b) occur in the paralogs. Each of these situations corresponds to an ordinary mutation producing an indel in one paralog, and this indel subsequently being transferred to the other paralog, or fixed, by gene conversion.

Cooperative shrinkage of paralogs can be quantified by phylogenetic detection of fixed conversion-induced indels (Figure 4). To perform this analysis, we ascertained all cases for which, ancestrally, two paralogs had identical lengths at a particular site and, in one sister, they acquired matching indels at that position. This condition implies that, in the ancestral lineage of the sister, ordinary mutation produced an indel in one paralog, and that this indel was later copied to the other paralog, or “fixed”, by gene conversion. In *Drosophila*, we detected 74 fixed insertions, with a total inserted sequence length of 391 nt, and 176 fixed deletions, with a total deleted sequence length of 1,660 nt. In primates, we detected four fixed insertions, with a total inserted sequence length of 4 nt, and 24 fixed deletions, with a total deleted sequence length of 438 nt. Thus, in both lineages, fixed deletions were much longer and more frequent than fixed insertions. Subtracting total insertion lengths from total deletion lengths, we arrived at effective deletion lengths of 1,269 nt in *Drosophila* and 434 nt in primates. The total sequence length of all paralogs was 208,956 nt in *Drosophila* and 4,916,824 nt in primates. Therefore, the shrinkage rate of paralogs by gene conversion is ∼0.11 per *K*_s_ unit in *Drosophila* and ∼0.015 per *K*_s_ unit in primates. This result implies that, in the absence of selection, these paralogs will exponentially shrink and disappear in ∼138 *K*_s_ units, or ∼6,210 million years, in *Drosophila* and ∼1,021 *K*_s_ units, or ∼612,600 million years, in primates.

## Methods

Whole-genome sequences of *Drosophila melanogaster*, *Drosophila simulans*, *Drosophila yakuba*, *Homo sapiens* (human), *Pan troglodytes* (chimpanzee), and *Pongo pygmaeus* (orangutan) were downloaded from the UCSC Genome Bioinformatics site at http://genome.ucsc.edu. We used Mega BLAST [25] (default parameters) and Bridges [26] (KM = 13, FilterDBase = 20, FilterQuery = 20, KS = 12, CoeffMis = 0.01, CoeffGap = 0.05, FlatGap = 10, MaxDist = 50, MinWeight = 100, CoeffMisPost = 0.1, MaxDistPost = 1000) to locate unique pairs of similar sequence segments (both coding and noncoding) in the genomes of *D. melanogaster* and *H. sapiens*. To avoid short repeats, we required that each sequence in a pair was greater than 100 nt long. After examining the output from these methods, we set a cutoff of 78% sequence identity between pairs of paralogs. If both paralogs were located on the same chromosome, we required that they were separated by greater than 100 nt to avoid sequencing or genome mapping errors. We used the BLASTN [27] (default parameters) and Mega BLAST (default parameters) algorithms to locate orthologs for each paralog in sister and outgroup species, using conserved synteny of 1,000 nt on either side of each sequence to ensure that orthologs were correctly assigned. Orthologs obtained via this method were verified using multiple species sequence alignments downloaded from the UCSC Genome Bioinformatics website (http://genome.ucsc.edu). To identify repetitive regions within paralogs, we ran RepeatMasker (http://www.repeatmasker.org) with cross_match (http://www.phrap.org) on human paralog sequences with a human-specific repeat library, and on *D. melanogaster* paralog sequences with a *Drosophila*-specific repeat library. Pairs of paralogs present in all three species of a lineage were aligned with MUSCLE [28] (default parameters), and alignments, particularly at indel positions, were checked by eye to ensure accuracy. Indels (both conversion-consistent and ordinary mutations) were removed from the analysis if they had different lengths or were located at either end of an alignment. We also excluded cases in which, at a particular position where an indel occurred in one sister, all other orthologous sequences were not identical. Meiotic recombination rates were obtained from the *Drosophila melanogaster* recombination rate calculator [29] for *D. melanogaster* and from the HapMap website at http://www.hapmap.org [30] for human. Statistical significance was determined with binomial sign tests for deletion biases, and paired t-tests for Spearman correlation coefficients. For each test, we used α = 0.05 and reported two-tailed probabilities.

## Supporting Information

Figure S1 Chromosomal distributions of paralogs in *D. melanogaster* (a) and human (b) genomes. Specific chromosomes are labeled on the x-axis, with “U” representing unmapped sequences. Plotted for each chromosome are distributions of its size in the genome (black bars), number of pairs of paralogs (gray bars), and number of pairs of paralogs that underwent gene conversion (blue bars).
(TIF)

Text S1 Supporting Methods.
(DOC)
