# Supplementary figures and images for: A Strong Deletion Bias in Nonallelic Gene Conversion
Source: PLoS Genet. 2012 Feb 16;8(2):e1002508. doi: 10.1371/journal.pgen.1002508 (PMC3280953; doi:10.1371/journal.pgen.1002508)

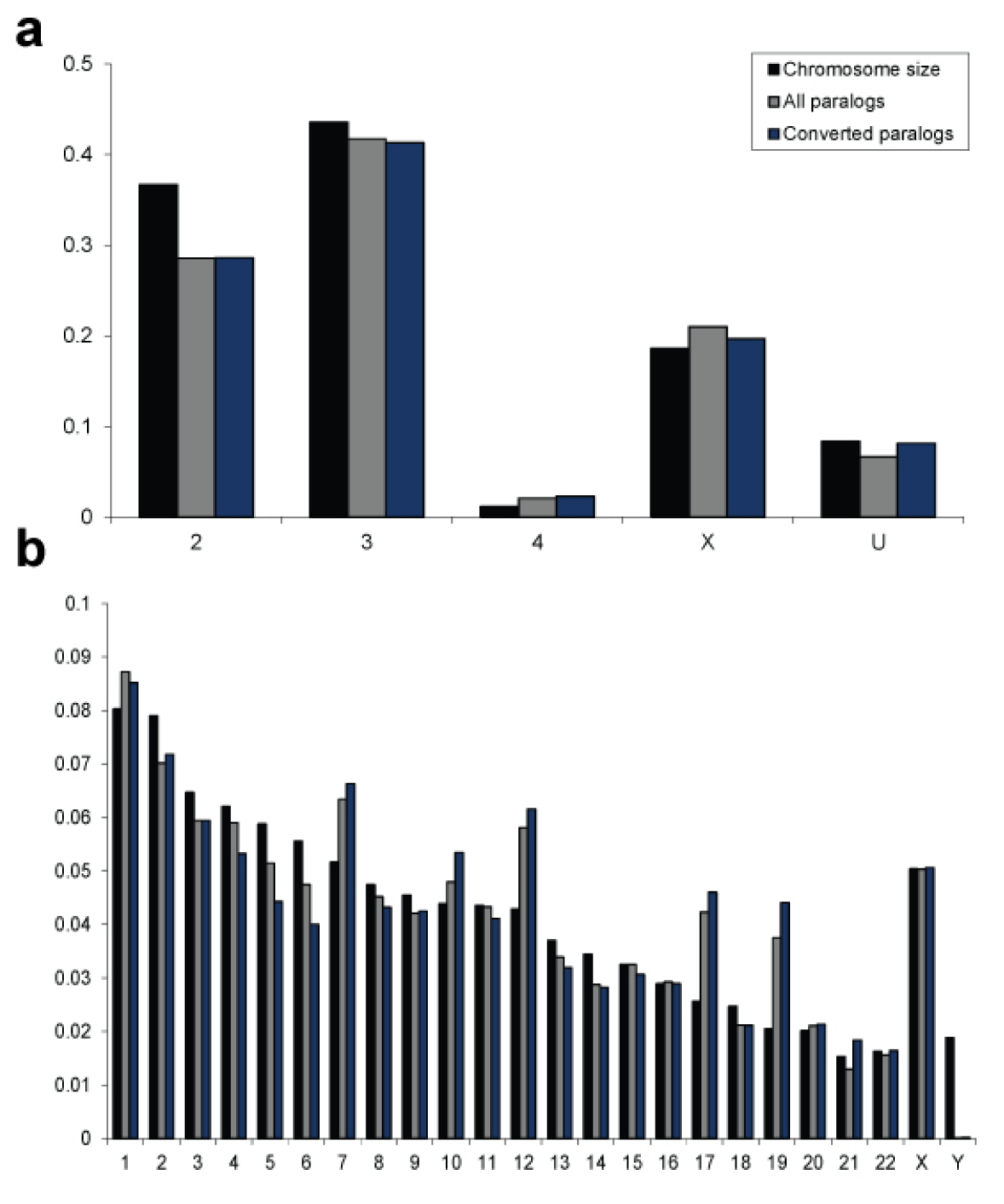

Supplement: Figure S1 — Chromosomal distributions of paralogs in D. melanogaster (a) and human (b) genomes. Specific chromosomes are labeled on the x-axis, with “U” representing unmapped sequences. Plotted for each chromosome are distributions of its size in the genome (black bars), number of pairs of paralogs (gray bars), and number of pairs of paralogs that underwent gene conversion (blue bars). (TIF) [file pgen.1002508.s001.tif]
