# Supplementary material for: A Strong Deletion Bias in Nonallelic Gene Conversion
Source: PLoS Genet. 2012 Feb 16;8(2):e1002508. doi: 10.1371/journal.pgen.1002508 (PMC3280953; doi:10.1371/journal.pgen.1002508)
Supplement: Text S1 — Supporting Methods. (DOC) [file pgen.1002508.s002.doc]

**Supporting Information**

**Supporting Methods**

***Estimating the proportion of gene conversion-consistent indels attributed to ordinary mutation***

It was important to assess the likelihood that some fraction of indels consistent with gene conversion were actually produced by ordinary mutations. To estimate the proportion of such false positives, we calculated the probability of observing ordinary mutations resembling gene conversion indels in each lineage. We considered the simplest case, in which there was an ancestral indel at a particular site in one paralog, and a second, identical, indel is produced by ordinary mutation at the same site in the second paralog. For simplicity, we assumed that each indel has a length of 1 nt. We denoted the probabilities that ordinary mutation generates an indel as *p*_indel_, that an indel generated by ordinary mutation is an insertion as *p*_i_ and a deletion as *p*_d_, and that an indel generated by ordinary mutation occurs at the same site as the first indel as *p*_site_. Given that the first indel was already present, the probability of arriving at such a configuration by ordinary mutation alone is *p*_ii_ = *p*_indel_ × *p*_i_ × *p*_site_ for insertions and *p*_dd_ = *p*_indel_ × *p*_d_ × *p*_site_ for deletions.

Assuming that insertions and deletions do not occur at the ends of sequences, as in our empirical analysis, and denoting the length of the second paralog as *l*, the probability that the second indel occurs at the same site as the first is *p*_site_ = 1/(*l*-1) if the first indel was an insertion and *p*_site_ = 1/(*l*-2) if it was a deletion.

We used *p*_indel_ = 3.42 × 10^−3^ for *Drosophila* [31], and *p*_indel_ = 0.03 for primates [32], which are the frequencies of lineage-specific indels observed in genomic data. For both lineages, we used *l* = 100 nt, which was the minimum paralog length in our dataset. There were 18 insertions and 184 deletions consistent with ordinary mutation in *Drosophila*, and 533 insertions and 562 consistent with ordinary mutation in primates. Thus, we used *p*_i_ = 0.09 and *p*_d_ = 0.91 for *Drosophila*, and *p*_i_ = 0.49 and *p*_d_ = 0.51 for primates.

In *Drosophila*, *p*_ii_  is ~3.11 × 10^−6^, and *p*_dd_ is ~3.18 × 10^−5^. In primates, *p*_ii_ is ~1.48 × 10^−4^, and *p*_dd_ is ~1.56 × 10^−4^. To estimate the number of such configurations in the *Drosophila* and primate datasets, we split each into *N* = *T*/*l* segments of length *l*, where *T* is the length of the target sequence on which the second indel mutation can occur (half of the total length of paralogs). Assuming that pairs of paralogs are independent, the probability that there are *m* mutations that fit the desired configuration in our dataset is binomially distributed. Thus, the expected number of such mutations is *Np*_ii_ for insertions and *Np*_dd_ fpr deletions. Applying this to our *Drosophila* dataset (*T* = 104,478), we expect to observe ~3.25 × 10^−3^ cases for insertions and ~0.03 cases for deletions. In primates (*T* = 2,458,412), these expectations were ~3.64 for insertions and ~3.84 for deletions. Thus, for this simple scenario, it is highly unlikely that more than a few conversion-consistent configurations were produced by ordinary mutation. Moreover, in both datasets, a substantial proportion of paralogs (Figure 2a) and indel events (Figure 3a) were larger than the values used for these calculations. Hence, the numbers of such configurations in our empirical datasets are even smaller than our estimates.

***Ascertaining the effect of sequencing errors on our observations***

In addition to ordinary mutation, some proportion of gene conversion-consistent indels may be artifacts of sequencing errors. To ensure that sequencing errors were not responsible for observed deletion biases, we calculated the proportions of insertions and deletions consistent with sequencing errors for each lineage. Because all observed indels can be due to sequencing errors, we conservatively assumed that the number of sequencing errors of each type (insertion or deletion) was the sum of conversion-consistent and ordinary mutation-consistent events of that type.

In *Drosophila*, we observed 197 insertions (179 conversions and 18 ordinary mutations) and 798 deletions (614 conversions and 184 ordinary mutations) that could be attributed to sequencing errors. In primates, there were 665 insertions (132 conversions and 533 ordinary mutations) and 1,017 deletions (455 conversions and 562 ordinary mutations) that could be due to sequencing errors. Because sequencing errors, like ordinary mutations, can occur at any site, the target size for such errors was 104,478 nt for *Drosophila* and 2,458,418 nt for primates (see above).

Dividing the sum of each type of indel by its target size yielded sequencing error-consistent insertion proportions of ~1.89 × 10^−3^ for *Drosophila* and ~2.7 × 10^−4^ for primates, and deletion proportions of ~7.64 × 10^−3^ for *Drosophila* and ~4.14 × 10^−4^ for primates. These small values imply that length difference sequencing errors are rare in our datasets.

For comparison, we also calculated the proportions of insertions and deletions consistent with gene conversion in each lineage. Because indels produced by gene conversion can only occur at sites in which there is an ancestral sequence difference, the target sizes for such events were 960 nt for *Drosophila* and 28,701 nt for primates. Given these targets, conversion-consistent insertion proportions are ~0.19 in *Drosophila* and ~4.6 × 10^−3^ in primates, and deletion proportions are ~0.64 for *Drosophila* and ~0.02 for primates. These proportions are all much higher than those of sequencing errors; therefore, only a very small proportion of observed indels can be artifacts of sequencing errors.

**Supporting References**

1. Begun DJ *et al.* (2007) Population Genomics: Whole-Genome Analysis of Polymorphism and Divergence in *Drosophila simulans*. PLoS Biol 5: e310.
2. The Chimpanzee Sequencing and Analysis Consortium (2005) Initial sequence of the chimpanzee genome and comparison with the human genome. Nature 437: 69–87.
